# The efficacy of adjunctive mitomycin C and/or anti-VEGF agents on glaucoma tube shunt drainage device surgeries: a systematic review

**DOI:** 10.1007/s00417-024-06476-z

**Published:** 2024-04-24

**Authors:** Raquel Figueiredo, Joao Barbosa-Breda

**Affiliations:** 1 Faculty of Medicine of the University of Porto, Porto, Portugal; 2 Department of Surgery and Physiology, UnIC@RISE, Faculty of Medicine of the University of Porto, Porto, Portugal; 3 Department of Ophthalmology, Centro Hospitalar E Universitário São João, Porto, Portugal; 4 Research Group Ophthalmology, Department of Neurosciences, KULeuven, Louvain, Belgium

**Keywords:** Glaucoma drainage implants, Mitomycin, Antimetabolites, Anti-vascular endothelial growth factor, Glaucoma

## Abstract

**Purpose:**

The effectiveness of mitomycin C (MMC) in trabeculectomy has long been established. The aim of this review is to evaluate the efficacy and safety of adjunctive agents in tube shunt drainage device surgery for glaucoma or ocular hypertension, since controversy still exists regarding their benefit.

**Methods:**

We searched CENTRAL, PubMed, Embase, Web of Science, Scopus, and BASE for RCTs, which have used adjuvant antimetabolites—either MMC or 5-Fluorouracil (5-FU)—and/or anti-vascular endothelial growth factors (anti-VEGF) agents. The main outcome was IOP reduction at 12 months.

**Results:**

Ten studies met our inclusion criteria. Nine used the Ahmed Glaucoma Valve (AGV) implant, while the double-plate Molteno implant was used in one study. Four studies used MMC. The remaining six studies used an anti-VEGF drug – either bevacizumab, ranibizumab or conbercept.

Only one MMC-study reported a significant difference in the IOP reduction between groups at 12 months, favouring the MMC group (55% and 51%; p < 0.01). A significant difference was also reported by two out of five bevacizumab-studies, both favouring the bevacizumab group (55% and 51%, p < 0.05; 58% and 27%, p < 0.05), with the highest benefit seen in neovascular glaucoma cases, especially when panretinal photocoagulation (PRP) was also used. Neither ranibizumab nor conbercept were found to produce significant differences between groups regarding IOP reduction.

**Conclusion:**

There is no high-quality evidence to support the use of MMC in tube shunt surgery. As for anti-VEGF agents, specifically bevacizumab, significant benefit seems to exist in neovascular glaucoma patients, especially if combined with PRP.


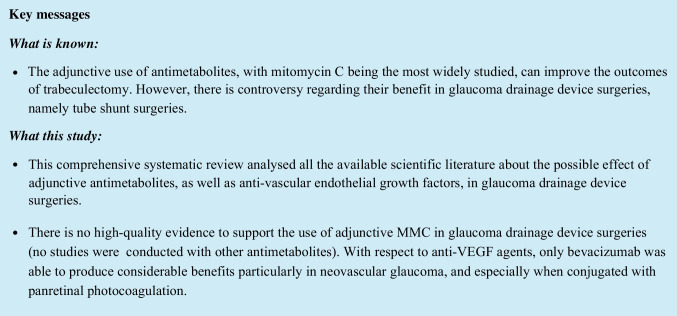


## Introduction

Glaucoma is a group of multifactorial and progressive optic neuropathies caused mainly by an intraocular pressure (IOP)-related damage to the retinal ganglion cells. Its origin is thought to lie in anatomical, vascular and/or genetic factors, among others [1].

Surgery plays an important role in the management of patients in whom medical therapy alone has failed to control disease progression [2]. Procedures such as trabeculectomy continue to be among the routinely used surgical approaches for glaucoma [3]. However, glaucoma drainage implants have been conquering their position in recent years, mainly after randomized trials have shown their comparative efficacy and safety [3, 4].

The implants more commonly used include the Ahmed glaucoma valve and the Ahmed ClearPath, the Molteno implant (Molteno Ophthalmic Limited, Dunedin, New Zealand), the Baerveldt glaucoma implant (Advanced Medical Optics, Santa Ana, California, USA) and the Paul glaucoma implant (PGI, Advanced Ophthalmic Innovations, Singapore, Republic of Singapore) [5]. These can be classified having into account the material in which they are made of (silicon or polypropylene, for example) and the type of opening, that is, valvular such as Ahmed, or non-valvular (as in all the remaining ones).

The fact that the Ahmed glaucoma device is a valvular implant accounts for its main difference when being compared with the other ones: it is associated with a better IOP control in the short-term, as its valvular mechanism helps preventing hypotony postoperatively [6].

The problem all these devices have in common is that postoperatively a fibrous capsule starts to form around the end plate, as part of a normal scarring process. It is this fibrous capsule around the end plate that offers the greatest resistance to aqueous flow across drainage implants, thus being the major limitating factor of their efficacy. Mitomycin C (MMC) has proven to increase the success of trabeculectomy (glaucoma surgery without device) due to its ability to modulate scarring. The same has been considered regarding tube shunt surgery, where it could modulate capsule thickness, by preventing fibroblast proliferation and lymphocyte activation-proliferation, the two main inflammatory cell types that lead to fibrosis [7, 8]. Another potential agent is 5-FU, that has also been used in trabeculectomy. Vascular endothelial growth factor is another important pharmacologically approachable target. The efficacy and safety of antimetabolites or anti-VEGF drugs as adjunctive agents in glaucoma tube shunt surgery is still controversial.

As far as we are concerned, this will be the first systematic review evaluating if mitomycin C and/or anti-VEGF agents’ use as adjunctive therapies in glaucoma drainage device implantation surgery can bring benefits to this type of glaucoma surgical approach as it does to other ones.

## Methods

This systematic review was conducted according to the Preferred Reporting Items for a Systematic Review and Meta-analysis (PRISMA) guidelines. Its protocol has been registered in PROSPERO (registration no. CRD42022292311).

### Search methods for identification of studies

We searched the following six electronic databases for randomized controlled clinical trials (RCTs) meeting the inclusion and exclusion criteria: Cochrane Central Register of Controlled Trials (CENTRAL), PubMed, Embase, Web of Science, Scopus, and Bielefeld Academic Search Engine (BASE), the latter being used for gray literature. No language or publication year restrictions were imposed. The electronic databases were last searched on April 27th 2022. The full search strategy for all databases can be found in the Supplemental Materials. We checked the reference lists of included trials for other studies fulfilling our inclusion criteria.

### Criteria for considering studies for this review

#### Types of participants

We included trials in which participants, previously diagnosed with any type of glaucoma or ocular hypertension in at least one eye, were submitted to surgery to implant one of the following tube shunt drainage devices: Ahmed glaucoma valve or Ahmed ClearPath (New World Medical, Rancho Cucamonga, California, USA), Molteno (Molteno Ophthalmic Limited, Dunedin, New Zealand), Baervedlt (Advanced Medical Optics, Santa Ana, California, USA), or Paul (PGI, Advanced Ophthalmic Innovations, Singapore, Republic of Singapore). We did not exclude trials in which participants had had previous ocular surgery or were undergoing tube shunt drainage device surgery simultaneously with other types of ophthalmic surgery (e.g. cataract surgery), whose effect on the outcomes will later be taken into consideration. No restrictions about demographic characteristics, such as age, gender, ethnicity, and comorbidities were applied.

### Type of intervention and comparison

We included RCTs which compared tube shunt drainage device surgery with and without the use of an adjuvant, which could be an antimetabolite – either MMC or 5-FU – or any anti-VEGF.

### Types of outcome measures

#### Primary outcome

The primary outcome was IOP reduction, evaluated as a relative reduction from baseline (preoperative IOP) at 12 months. Data from months 6 and 9 were also considered whenever significant.

### Secondary outcomes

1. Mean IOP at baseline and at 12 months, measured using Goldmann applanation tonometry or another standard device.
2. Mean number of glaucoma medications taken at baseline and after 12 months (regardless of the route of administration)
3. The rates, expressed in percentage, of the most commonly occurring intra- and postoperative complications.
4. Mean best corrected visual acuity at baseline and at 12 months, measured using the LogMAR scale.

### Follow-up

We placed no restriction on the duration of follow-up of included studies. However, the primary analysis of outcomes was at 12 months after surgery.

### Criteria for excluding studies from this review

Studies were excluded if they met at least one of the following exclusion criteria: (1) non-human subjects (animal studies); (2) full text or data regarding the primary outcome not available, even after trying to contact the original authors. No language restrictions were imposed.

### Data collection and analysis

#### Selection of studies

All titles and abstracts identified in the search were screened by two authors (RF, JBB) independently of one another in order to assess if inclusion criteria were met. In case of disagreement, the more qualified review team member's judgment prevailed. Finally, all studies that met the inclusion criteria underwent data extraction and were assessed for risk of bias by the main author (RF), under the supervision of a senior one (JBB).

#### Assessment of risk of bias in included studies

We assessed all included studies for potential risk of bias according to methods presented in the Cochrane Handbook for Systematic Reviews of Interventions [9]. Five domains were evaluated: method of sequence generation and concealment of allocation before randomization (selection bias), masking of investigators and participants (performance bias), masking of outcome assessors (detection bias), rates of follow-up and intention-to-treat analysis (attrition bias), selective outcome reporting (reporting bias), and other potential sources of bias, such as funding sources. Each study was catalogued as “low risk”, “unclear risk”, or “high risk” with respect to each risk of bias type, based on the available information. Furthermore, we used the second version of the Cochrane risk-of-bias tool for randomized trials (RoB 2) [10], which is structured into a fixed set of domains of bias.

#### Data extraction and management

Details of study design and methods, participant characteristics, and primary and secondary outcomes were extracted onto standardised data charting forms. Key data extracted from each article included: phakic status and glaucoma type; tube placement location; the class of the adjuvant pharmacological therapy that was used in each RCT, as well as its dose and timing of administration; the pre- and post-operative IOPs, intra- and postoperative complication types and rates, class and number of IOP-lowering medications; and finally, the need for additional glaucoma surgeries. Original authors were contacted in order to provide complementary data.

### Data synthesis

Narrative synthesis of evidence was undertaken for all included studies. Our results describe participants’ characteristics considered to be important for outcomes’ appreciation, such as their phakic status, glaucoma subtypes, and the class, dose and timing of administration of the adjuvant pharmacological interventions. Our results focus on the relative IOP reduction achieved with each surgery type, the mean number of glaucoma medications and the type and rates of the most prevalent postoperative complications.

## Results

### Description of studies

**Fig. 1:**
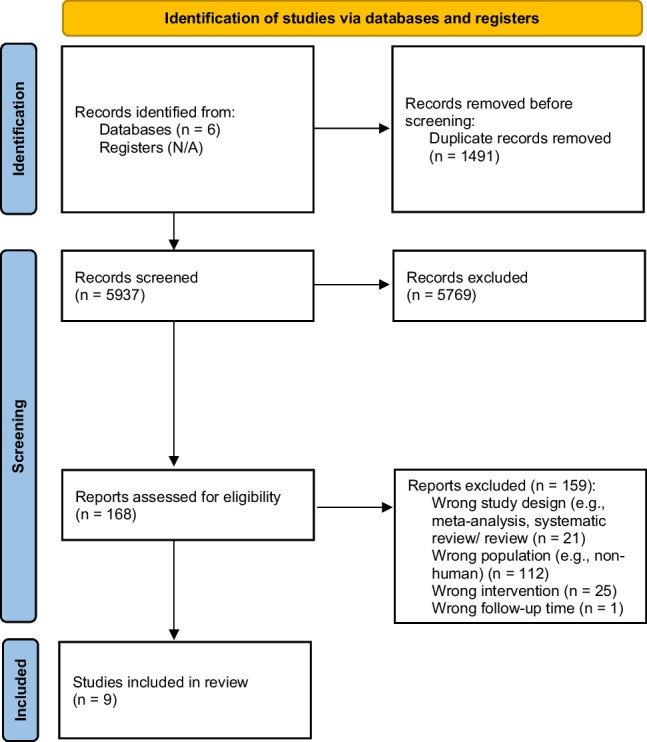
PRISMA flowchart diagram for study identification and selection. This flowchart visually summarises the studies identification and selection process

**Fig. 2:**
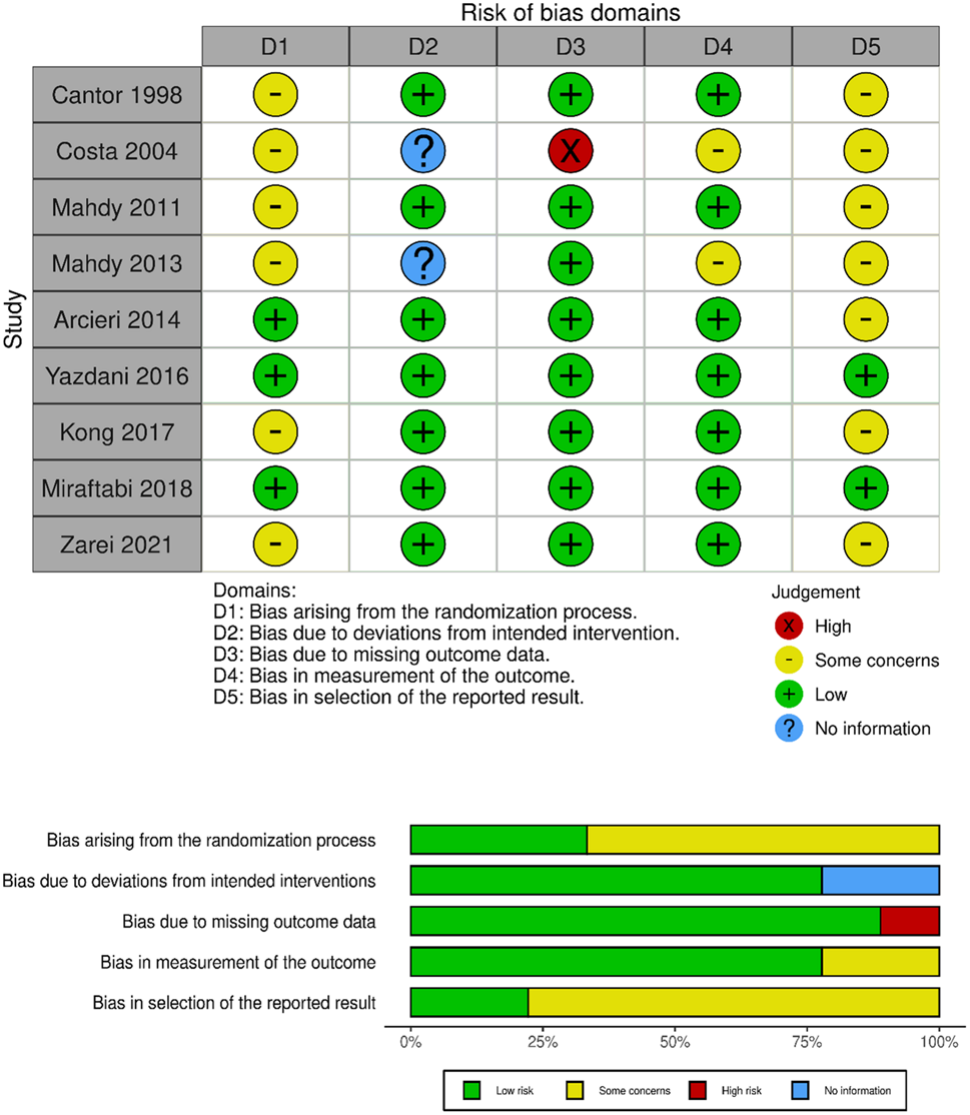
Methodological quality summary (Cochrane risk of bias assessment tool for randomized trials): review authors’ judgements about each methodological quality item for each included study. Only Arcieri et al. (2014) [36], Yazdani et al. (2016) [37] and Miraftabi et al. (2018) [38] explicitly reported the randomization method. In the study performed by Costa et al. (2004) [11], 28/60 (47%) participants did not complete one year of follow-up. Only Yazdani et al. (2016) and Zarei et al. (2021) [12] published a protocol or a trial registry record to compare outcomes with an official source

The electronic search strategy resulted in a total of 7428 studies. After removing duplicates, title and abstract screening and full-text screening, 9 studies met our criteria (Fig. 1), comprising a total of 453 eyes of 441 patients with glaucoma. Studies are well geographically distributed: 3 from Iran, 2 from Brazil and Egypt and 1 from USA and China (Fig. 2).

### Types of participants

All included participants had been diagnosed with diverse types of glaucoma (both primary and/or secondary) and had an IOP that was not controlled with maximum tolerated medical therapy. No included studies were conducted in patients with ocular hypertension.

Seven of the 9 trials had 2 study arms (intervention and control/placebo), while two had 3 study arms (2 interventions) (Table 1). Eight out of 9 studies used the Ahmed Glaucoma Valve (AGV) implant. Cantor et al. (1998) [11] was the exception, where the double-plate Molteno implant was used.

**Table 1:** Characteristics of included studies. Demographic characteristics of the study participants

Author and year		No. pts	No. eyes	Age (mean ± SD) (yrs)	Follow-up (mean ± SD)	Types of glaucoma	Type of device		Intervention(s)	Comparator	Timing of adjunctive application
Cantor 1998	MMC	12	12	66.4 ± 14	N/A	N/A	DPM	N/A	MMC, 0.4 mg/ml, 2 min	Placebo: BSS sponge, 2 min	Intraoperatively, immediately prior to DPM implantation
	BSS	13	13	67.2 ± 11.5	N/A						
Costa 2003	MMC	34	34	62 ± 16.5	12.3 ± 6.7 mo	POAG, NVG, aphakic/pseudophakic, IFG, traumatic, CACG, post-PK, steroid-induced	AGV	N/A	MMC, 0.5 mg/ml, 5 min	Placebo: BSS sponge, 5 min	Intraoperatively, immediately prior to AGV implantation
	Control	26	26	61.1 ± 20.6	13.5 ± 7.0 mo						
Mahdy 2011	MMC	14	20	4 ± 5	12 mo	PCG	AGV	New World Medical, Inc, Rancho Cucamonga, CA	MMC, 0.4 mg/ml, 3 min OR Bevacizumab, 1.25 mg/0.05 ml	No placebo	MMC intraoperatively, immediately prior to AGV implantation; bevacizumab after surgery completion
	Bev	16	20	5 ± 5	12 mo						
	Control	18	20	5 ± 4	12 mo						
Mahdy 2013	Bev	20	20	55 ± 1.3	16.2 ± 2.6 mo	NVG	AGV	New World Medical Inc., Rancho Cucamonga, CA	IV Bevacizumab, 1.25 mg/0.5 ml, and PRP	AGV plus PRP	IVB 2 weeks before AGV implantation
	Control	20	20	56 ± 4.3	16.8 ± 1.2 mo						
Arcieri 2014	Bev	20	20	59.25 ± 8.05	2.15 ± 0.67 yrs	NVG	AGV	New World Medical Inc, Rancho Cucamonga, CA, USA	IV Bevacizumab, 1.25 mg	No injection	Intraoperatively at the end of surgery, and 4 and 8 weeks after surgery
	Control	20	20	62.40 ± 11.78	2.35 ± 0.67 yrs						
Yazdani 2016	MMC	25	25	36.6 ± 20.6	51.3 ± 3.8 w	Aphakic, pseudophakic, NVG, DG, IFG, PCG, JOAG, POAG, CACG, PEXG, GCG, traumatic, steroid-induced	AGV	Model FP7; New World Medical Inc., Rancho Cucamonga, CA	MMC, 0.02 mg/ml, 3 min	No placebo	Intraoperatively, immediately prior to AGV implantation
	Control	25	25	33.3 ± 20.1	52.4 ± 4.2 w						
Kong 2017	Ranib	26	26	53.00 ± 12.32	N/A	NVG	AGV	FP7	IV Ranibizumab, 10 g/l OR IV Conbercept, 10 g/l	No placebo	IVR or IVC 3–7 days before AGV implantation
	Conb	21	21	54.00 ± 12.74	N/A						
	Control	21	21	54.24 ± 11.05	N/A						
Miraftabi 2018	Bev	25	25	51.36 ± 15.44	10.56 ± 2.61 mo	POAG, CACG, PEXG, PCG, post-PK, post-vitrectomy	AGV	FP7, New World Medical	SC Bevacizumab, 2.5 mg/0.1 ml	No placebo	At the site of the shunt plate, after suturing the conjunctiva
	Control	25	25	58.76 ± 12.11	10.32 ± 2.74 mo						
Zarei 2021	Bev	30	30	56.7 ± 17.2	N/A	NVG, POAG, PEXG, PCG, post-PK, uveitic, post-vitrectomy, post-DSEK, aphakic	AGV	N/A	SC Bevacizumab, 1.25 mg	No injection	Around the shunt plate, at the end of surgery
	BSS	30	30	57.4 ± 16.4	N/A						

Studies are ordered according to date of publication. AGV: Ahmed glaucoma valve; BSS: balanced salt solution; CACG: chronic angle-closure glaucoma; DG: developmental glaucoma; DPM: double-plate Molteno; GCG: ghost cell glaucoma; IFG: inflammatory glaucoma; IVB: intravitreal bevacizumab; IVC: intravitreal conbercept; IVR: intravitreal ranibizumab; JOAG: juvenile open-angle glaucoma; MMC: mitomycin C; NVG: neovascular glaucoma; PCG: primary congenital glaucoma; PEXG: pseudoexfoliative glaucoma; PG: pigmentary glaucoma; POAG: primary open-angle glaucoma; Post-DSEK: post-descemet stripping endothelial keratoplasty; Post-PK: post-penetrating keratoplasty; PRP: panretinal photocoagulation; SC: subconjunctival

Four studies used MMC as adjuvant. Its concentration and time of application ranged from 0.4 to 0.5 mg/mL and from 2 to 5 min. The remaining five studies used a type of anti-VEGF drug, either intravitreal or subconjunctival. One of such drugs was bevacizumab (1.25 mg in 0.05 mL or 2.5 mg in 0.1 mL), in administration time points that ranged from 2 weeks before to 8 weeks after Ahmed glaucoma valve (AGV) implantation surgery. One study used other anti-VEGF drugs as adjuvant*s*. Ranibizumab was compared to conbercept and to AGV without any adjuvant in a Chinese study performed by Kong et al. (2017) [12]. No studies were conducted with 5-fluoruracil.

Four studies included patients who had been diagnosed with primary open-angle glaucoma (POAG), while 3 studies included patients with chronic angle-closure glaucoma (CACG). Neovascular glaucoma (NVG) was included in 6 studies; aphakic/pseudophakic glaucoma and pseudoexfoliative glaucoma (PEXG) were included in 5 and 4 studies, respectively; glaucoma after procedures such as penetrating keratoplasty (post-PK), vitrectomy and Descemet’s stripping endothelial keratoplasty (post-DSEK) were less commonly included: in 3, 2, and 1 studies, respectively; inflammatory glaucoma (IFG) was included in 3 studies; other types of secondary open-angle glaucoma (SOAG) were steroid-induced and traumatic glaucoma (included in 2 studies each), and ghost cell glaucoma (included in only 1 study). 3 studies included primary congenital glaucoma (PCG) and one included juvenile open-angle glaucoma (JOAG).

None of the studies reported statistically significant differences between the two groups of participants, in terms of their demographic and preoperative characteristics, except for Cantor et al. (1998) and Zarei et al. (2021) who respectively outlined that the MMC group did not include any black patients (that represented 23% of the control group) and that the pre-operative visual acuity was higher in the AGV-only group (mean LogMAR visual acuity scores of 0.78 and 1.29; p = 0.008).

Mahdy et al. (2011) [13] chose a remarkably different population from all the other studies, namely in terms of their age, which ranged from 1 to 6 years old, with a mean of 5 ± 5.3 years. All other studies were conducted with adult participants.

Regarding previous surgeries, Cantor et al. (1998) included those with any previous ocular surgery other than scleral buckling; Mahdy et al. (2011) only enrolled patients who had had a prior failed trabeculotomy or goniotomy (which are generally the preferred surgical approach for primary congenital glaucoma); Ariceri et al. (2014) included patients with uncontrolled neovascular glaucoma (NVG) who had already undergone PRP and still required glaucoma drainage device implantation; and Zarei et al. (2021) excluded those with previous shunt surgery or cyclodestructive procedure.

Furthermore, Cantor et al. (1998) admitted the possibility of undergoing cataract extraction within the follow-up period or even simultaneously with the drainage device surgery, whereas Yazdani et al. (2016) and Zarei et al. (2021) excluded patients who were submitted to any additional procedure at the time of AGV implantation.

Cantor et al. (1998) and Costa et al. (2004) included patients undergoing glaucoma drainage device implantation – double-plate Molteno implant surgery and AGV implantation, respectively – regardless of their lens status (thereby including phakic, pseudophakic and aphakic patients), while Mahdy et al. (2011) excluded patients with aphakic glaucoma.

The remaining included studies do not explicitly mention additional information regarding which prior surgeries were allowed or if simultaneous cataract surgery was allowed.

### IOP-lowering efficacy

Regarding the 4 studies that used MMC as adjuvant, only Mahdy et. al (2011) reported a significant difference between groups at 12 months of follow-up, with higher IOP values in the control group. IOP was significantly higher in the control group in comparison with the MMC group (p < 0.01), after decreasing 51% and 55% in each group, respectively, when compared with their preoperative values. This was the only included study conducted in children. Pairwise comparisons made by Cantor et al. (1998) to investigate whether MMC offers any clinical advantage to double-plate Molteno implant surgery showed that its use did not significantly reduce IOP more than the use of a placebo (p value not reported) (Table 2). The authors also concluded that the only factor significantly affecting IOP is the time after surgery, since IOP was the lowest one week after surgery and then progressively increased with time. Costa et al. (2004) also reported no significant differences between groups at all follow-up time intervals, except on the 7th and 15th days after surgery, when they were significantly lower in the MMC group (p = 0.025 and p = 0.021, respectively), leading the authors to the conclusion that MMC is only partially successful during the initial wound healing process. Yazdani et al. (2016) also reported no significant differences at 12 months of follow-up. However, mean IOP was lower in the MMC group during the first month after surgery, reaching a significant difference at the third postoperative week (p = 0.04). IOP then returned to comparable values at all postoperative time intervals afterwards, including at 12 months (absolute values at each time point not reported).

**Table 2:** Summary of findings for mean IOP and number of glaucoma medications relative reduction

Study and year	Group	Preoperative mean IOP ± SD (mmHg)	12 mo mean IOP ± SD (mmHg)	IOP reduction (%)	Mean no. of preoperative medications ± SD (No.)	Mean no. of postoperative 12 months medications ± SD (No.)
Cantor 1998	MMC	41.5 ± 5.1	15.6 ± 3.4	62	3 ± 0.25	0.8 ± 0.25
	BSS	34.7 ± 2.3	15.3 ± 2.3	56	3 ± 0.34	1.1 ± 0.28
p value		> 0.05	> 0.05	> 0.05	> 0.05	> 0.05
Costa 2003	MMC	31.6 ± 9.5	15.1 ± 4.0	52	3.2 ± 0.8	1.3 ± 1.0
	Control	35.4 ± 10.1	15.3 ± 3.5	57	2.9 ± 1.1	1.3 ± 0.8
p value		0.141	0.864	> 0.05	0.190	0.961
Mahdy 2011	MMC	32 ± 3.5	14.5 ± 1.5	55	N/A	N/A
	Bevacizumab	33.6 ± 4.9	15 ± 2	55	N/A	N/A
	Control	35 ± 4.0	17 ± 3.1	51	N/A	N/A
p value		> 0.05	** < 0.01**	< 0.05	N/A	N/A
Mahdy 2013	Bevacizumab	38.4 ± 4.7	16 ± 7	58	N/A	N/A
	Control	38.5 ± 7.5	28 ± 8.4	27	N/A	N/A
p value		0.053	** < 0.01**	< 0.05	N/A	N/A
Arcieri 2014	Bevacizumab	40.10 ± 13.33	17.40 ± 9.99	58	2.85 ± 1.18	1.21 ± 1.12
	Control	38.35 ± 10.34	16.00 ± 3.98	57	2.80 ± 0.69	1.18 ± 0.73
p value		0.6454	0.4598	> 0.05	0.3912	0.9106
Yazdani 2016	MMC	32.1 ± 9.9	N/A	N/A	3.5 ± 0.8	0.65; 95% CI, 0.44–0.86
	Control	31 ± 9.4	N/A	N/A	3.6 ± 0.7	0.72; 95% CI, 0.56–0.88
p value		0.866	0.30	N/A	0.232	0.22
Kong 2017	Ranibizumab	45.13 ± 8.94	21.08 ± 3.15	53	3.12 ± 0.32	1.31 ± 0.79
	Conbercept	44.39 ± 10.62	21.00 ± 3.80	53	3.10 ± 0.30	1.38 ± 0.92
	Control	44.11 ± 9.38	23.14 ± 4.75	48	3.14 ± 0.36	1.62 ± 0.80
p value		0.93	> 0.05	> 0.05	0.90	> 0.05
Miraftabi 2018	Bevacizumab	27.52 ± 8.57	14.00 ± 3.52	49	3.48 ± 0.65	1.42 ± 0.90
	Control	24.88 ± 7.62	15.72 ± 4.86	37	3.32 ± 0.80	1.83 ± 0.92
p value		0.25	0.22	> 0.05	0.545	0.167
Zarei 2021	Bevacizumab	35.3 ± 10.5	17.6 ± 5	50	3.7 ± 0.5	2.1 ± 1.4
	BSS	32.4 ± 10.6	18.4 ± 2.5	43	3.7 ± 0.4	2 ± 1.1
p value		0.293	0.185	> 0.05	0.576	0.842

Studies are ordered according to date of publication. BSS: balanced salt solution; IOP: intraocular pressure; MMC: mitomycin C. IOP reduction (%) was calculated by the authors of this systematic review

Regarding bevacizumab, 2 studies out of 5 studies reported significant differences between groups after 12 months of follow-up. Arcieri et al. (2014) randomized patients with uncontrolled NVG that had already undergone PRP and still required glaucoma drainage device implantation to either receive 1.25 mg of intravitreal bevacizumab (IVB) or no injection, during Ahmed valve implant surgery. They reported no significant differences at 12 months of follow-up (p > 0.2497). However, at the 18th month postoperative follow-up visit, the IVB group had a significantly lower mean IOP than the control group (14.57 ± 1.72 mmHg and 18.37 ± 1.06 mmHg; p = 0.0002), and at 2 years a trend was noted in the same direction (14.43 ± 0.53 mmHg and 16.67 ± 4.40 mmHg; p = 0.0526). The authors conclude by suggesting that there is a trend to slightly lower IOPs with IVB use during AGV implantation for neovascular glaucoma at 2 years. Miraftabi et al. (2018) compared 2.5 mg of subconjunctival bevacizumab with surgery without adjuvant and found no significant differences between groups after 12 months of follow-up (p = 0.83). However, they report significant differences at the 6th month of follow-up (IOP was 14.12 ± 4.0137 in the bevacizumab group and 16.52 ± 4.29 in the control group; p = 0.04). More recently, Zarei et al. (2021) compared 1.25 mg of subconjunctival bevacizumab with 0.1 mL of normal saline and concluded that there were no significant differences between groups after one year of follow-up (p = 0.185). Nonetheless, they report significant differences at the 3rd month of follow-up (after a reduction from 35.3 ± 10.5 to 17.3 ± 6.2 mmHg and from 32.4 ± 10.6 to 20.7 ± 4.6 mmHg; p = 0.04). On the other hand, Mahdy et al. (2013) [14] submitted 40 patients with refractory NVG to AGV implantation plus PRP, with or without a single IVB injection performed 2 weeks prior to surgery and reported significantly lower IOP values in the bevacizumab group in comparison with the control group at 12 months (p < 0.05). At the 18th month postoperative follow-up visit, the bevacizumab group also had a significantly lower mean IOP than the control group (16 ± 4.2 mmHg and 28 ± 6.5 mmHg; p < 0.01). Mahdy et al. (2011) also reported significantly lower IOP values in the bevacizumab group in comparison with the control group at 12 months (p < 0.05), in their 3-armed study in which MMC was also included. IOP decreased 55% in both bevacizumab and MMC groups and 51% in the control group.

As for the other anti-VEGF agents, Kong et al. (2017) used a 3-armed study to compare ranibizumab to conbercept intravitreal injection (both given 3 to 7 days before AGV implantation) and to AGV alone for the treatment of neovascular glaucoma. There were no significant differences between groups after 12 months of follow-up (p > 0.05).

### Secondary outcomes

#### Number of glaucoma medications

Regarding the 4 studies that used MMC, only 3 reported this outcome. Mahdy et al. (2011) did not report this parameter. There were no significant differences between groups in the mean number of glaucoma medications at 12 months in the studies performed by Cantor et al. (1998), Costa et al. (2003) and Yazdani et al. (P > 0.05, P = 0.961 and P = 0.22, respectively). Yazdani et al. (2016) also approached this outcome in a slightly different manner, resorting to the cumulative survival of medication-free IOP control, i.e., % patients remaining free of IOP lowering drops, during the follow-up period. They reported no significant differences between groups. Medical therapy was initiated in 16 (64%) eyes in the MMC group and 19 (82%) eyes in the control group (P = 0.30) at a median of 12 (95% CI, 7.4–16.6) and 6 (95% CI, 4.4–7.5) weeks after surgery (P = 0.13).

Regarding the 5 studies that used bevacizumab, only 3 reported this outcome. Mahdy et al. (2011 & 2013) opted not to measure this parameter. There were no significant differences between groups in the mean number of glaucoma medications at 12 months in the studies performed by Arcieri et al. (2014), Miraftabi et al. (2018) and Zarei et. al (2021) (p > 0.910, p = 0.167 and P = 0.842, respectively). Remarkably, Arcieri et al. (2014) reported a trend for patients treated with bevacizumab to use less medication than the control group 24 months after surgery (with a p value of 0.0648).

Concerning ranibizumab and conbercept, Kong et al. (2017) reported no significant differences between these groups and the control group in the mean number of glaucoma medications at 12 months.

### Complications

Regarding the 4 studies that used MMC as adjuvant, there were no significant differences between groups in complication rates (Table 3).

**Table 3:** Most commonly occurring intra- and postoperative complications’ rates, expressed in percentage, and by group, and the respective p values

Study and year	Group	Choroidal effusion No. (%)		Flat anterior chamber No. (%)		Hypotony No. (%)			Retinal detachment No. (%)		Hyphema No. (%)	
Cantor 1998		Early	Late	Early	Late	Early		Late	Early	Late	Early	Late
	MMC	1 (< 1)	N/A	5 (42)	N/A	7 (50)		1 (< 1)	N/A	1 (< 1)	3 (25)	N/A
	BSS	3 (23)	N/A	2 (15)	N/A	5 (38)		0	N/A	0	4 (31)	N/A
	p value	> 0.05	> 0.05	> 0.05	> 0.05	> 0.05	> 0.05		> 0.05	> 0.05	> 0.05	> 0.05
Costa 2003	MMC	6 (18)		1 (3)		0			1 (3)		N/A	
	Control	5 (19)		2 (8)		1 (4)			1 (4)		N/A	
	p value	1.000		0.573		0.433			1.000		N/A	
Mahdy 2011	MMC	6 (30)		6 (30)		N/A			N/A		2 (10)	
	Bevacizumab	4 (20)		8 (40)		N/A			N/A		0	
	Control	4 (20)		8 (40)		N/A			N/A		2 (10)	
	p value	> 0.05		> 0.05		> 0.05			> 0.05		> 0.05	
Mahdy 2013	Bevacizumab	1 (5)		5 (25)		2 (10)			N/A		4 (20)	
	Control	2 (10) of which 1 (5) SCH		6 (30)		3 (15)			N/A		17 (85)	
	p value	> 0.05		> 0.05		> 0.05			> 0.05		> 0.05	
Arcieri 2014	Bevacizumab	3 (15)		2 (10)		N/A			0		2 (10)	
	Control	4 (20)		1 (5)		N/A			1 (5)		6 (30)	
	p value	1.0000		1.0000		N/A			1.0000		0.2351	
Yazdani 2016	MMC	2 (8)		N/A		N/A			N/A		2 (8)	
	Control	0 (0)		N/A		N/A			N/A		4 (17)	
	p value	> 0.05		> 0.05		> 0.05			> 0.05		> 0.05	
Kong 2017	Ranibizumab	N/A		7 (27)		3 (12)			N/A		11 (42)	
	Conbercept	N/A		6 (29)		3 (14)			N/A		9 (43)	
	Control	N/A		5 (24)		2 (10)			N/A		10 (48)	
	p value	N/A		0.94		0.89			N/A		0.93	
Miraftabi 2018	Bevacizumab	0		3 (12)		N/A			N/A		4 (16)	
	Control	2 (8)		3 (12)		N/A			N/A		0	
	p value	> 0.05		> 0.05		> 0.05			> 0.05		> 0.05	
Zarei 2021	Bevacizumab	2 (6.7)		3 (10)		N/A			N/A		11 (36.7)	
	BSS	6 (20)		12 (40)		N/A			N/A		10 (33.3)	
	p value	0.254		**0.007**		N/A			N/A		0.787	

It is noted that the only statistically significant difference concerns flat anterior chamber rates, that was significantly higher in control group than in bevacizumab one

Studies are ordered according to date of publication. BSS: balanced salt solution; MMC: mitomycin C; N/A: not applicable

However, Cantor et al. (1998) emphasized the fact that flat anterior chamber (AC), suprachoroidal hemorrhage (SCH), and “early” hypotony, defined by the authors as “early onset complications” (less than one month postoperatively), were more commonly seen in the MMC group (42% versus 15%; 17% versus < 1%, and 50% versus 38%; p values not reported), which could mean that there was a clinically significant difference despite absence of statistical significance (p values not reported). In likely manner, late postoperative complications, in which persistent hypotony and “SCH/choroidals” are included, were not considered to be significantly different between groups.

Concerning bevacizumab’s potential impact on postoperative complication rates, only Zarei et. al (2021) reported a significant difference. In this study, shallow AC was the highest overall complication, with a significantly higher rate of 40% (12 cases) in the control group versus 10% (3 cases) in the bevacizumab group (P = 0.007). All other complications were not significantly different between groups. The other four studies that used bevacizumab reported no significant differences between groups. In fact, Arcieri et al. (2014) did not report any major intra- and/or postoperative complication in either group, nor any significant difference between them. However, the authors highlighted the significantly lower extension(s) of new vessels in the anterior chamber angle and iris surface in the bevacizumab group (P = 0.0017 and P = 0.0015). Likewise, in Mahdy et al. (2013), the bevacizumab group showed a marked regression of iris and retinal neovascularization [in 14 eyes (70%) there was complete regression of neovascularization].

As for intravitreal ranibizumab and conbercept, Kong et al. (2017) found no significant differences in ocular or systemic adverse effects between groups, by registering very similar incidences of all the observed postoperative complications.

### Visual acuity

Only two studies [Cantor et al. (1998) & Miraftabi et al. (2018)] provided BCVA values at baseline and 12 months. In Cantor et al. (1998), VA changed from 1.0 ± 0.2 to 1.5 ± 0.4 logMAR in the MMC group and from 1.1 ± 0.2 to 2.1 ± 0.5 in the control group. As for Miraftabi et al. (2018), VA changed from 0.85 ± 0.85 to 1.00 ± 1.11 in the bevacizumab group and from 0.94 ± 0.79 to 0.87 ± 0.83 in the control group. Of these, only Cantor et al. (1998) compared VA values between groups at 12 months, by reporting that there was no significant difference in VA change between groups (p value not reported).

In fact, only 5 studies reported a statistical analysis comparing VA values between groups at 12 months [Cantor et al. (1998), Cantor et al. (2014), Kong et al. (2017), Miraftabi et al. (2018) and Zarei et al. (2021)]. Of these, none reported a significant difference.

### Risk of bias in included studies

Only two studies [Yazdani et al. (2016) and Miraftabi et al. (2018)] were classified as being at low risk of bias. On the other hand, two studies [Costa et al. (2004) and Mahdy et al. (2013)] were graded at high risk of bias (ie. because i) there was a single judgement of high risk of bias within any partial domain or ii) there were concerns in multiple domains [9]).

## Discussion

This is the first systematic review regarding the use of adjuvants in glaucoma tube shunt surgery. Overall, no (clinically) significant differences seem to exist with the use of any type of adjuvant (MMC or anti-VEGF agents), except for anti-VEGF agents (more specifically bevacizumab) in neovascular glaucoma patients [15–18].

On the other hand, MMC is effective in improving bleb survival after trabeculectomy. A Cochrane systematic review including 698 participants showed a reduction of surgical failure rates, even in the high-risk of failure group of participants [19]. Contradictory data has been published to determine if the same benefit exists in tube shunt surgery, as well as with other potential adjuvants, such as anti-VEGF agents. Thence, this systematic review describes the current evidence available for the impact of adjunctive MMC and/or anti-VEGF agents on tube shunt surgery.

As previously stated, out of 4 studies using MMC, only Mahdy et al. (2011) reported a significant difference between groups at 12 months of follow-up, which favoured the MMC group. Nonetheless, it may be argued that this may not be clinically meaningful since there was an IOP reduction of 55% (from 32 ± 3.5 to 14.5 ± 1.5 mmHg) in the MMC group and of 51% (from 35 ± 4.0 to 17 ± 3.1 mmHg) in the control one. The only major difference between this study and the other MMC ones lies in its population, since all the included patients were younger than 6 years of age, with an equal age distribution between them. This could possibly be explained by a stronger inflammatory (healing) response in infancy, which would give antifibrotic agents such as MMC more substrate to act. It would be important to perform RCTs directly comparing MMC results in an adult and child population in order to prove this difference [20–23]. Other important aspects that could potentially explain these results, such as the technique of AGV implantation and MMC dose and timing of administration were not different between this and the other MMC studies. Despite the fact that MMC has not been associated with any additional complication when compared to the group in which it was not applied, in view of the small difference between groups, we question the benefit of the routine incorporation of MMC in these surgical procedures.

Despite none of the other studies reported significant differences in IOP reduction at 12 months, two studies reported a significant difference at earlier follow-up times, specifically in the 7th and 15th days after surgery, and in the third postoperative week. Hence, one can allege that this early sharper IOP decrease more commonly seen in the MMC group was the result of some early effect on initial wound healing that, in fact, is not sustained over time [11]. The presence of the implanted drainage device, which technically can be understood as a foreign body, and consequently a constant stimulus for the immune system, with inflammatory cells’ migration and fibroblastic proliferation, may supersede the antimitotic effects of MMC [23–25] (this hypothesis would also explain the difference between the long-term results of MMC in trabeculectomy *versus* aqueous shunt surgery). Cantor et al. (1998) also suggested that a possible explanation for the early greater IOP reduction in the MMC group is related to the toxic effect this medication has on the ciliary body epithelium, which could, in combination with postoperative inflammation, reduce aqueous humour production; subsequently, as the postoperative inflammation subsided and ciliary body function was restored, the IOP would begin to rise. Overall, this early sharper IOP reduction does not alter the long-term (12 months) IOP (taking into account that there were also no significant differences in terms of IOP lowering medications), and as such it does not seem to outweigh the potential adverse effects MMC may have. Furthermore, the 4 studies using MMC applied concentrations that ranged from 0.02 to 0.5 mg/ml, for 2 to 5 min. Different doses and application times make accurate comparisons of these studies difficult but can provide us with a comprehensive range of its potential effects. Moreover, surgical procedures are thought to have been conducted in a sufficiently homogenous way, so that they cannot influence our results.

Out of the 6 studies using anti-VEGF agents, only 2 [Mahdy et al. (2011 and 2013)] reported a significant difference between groups at 12 months of follow-up, which favoured the bevacizumab group over the control one. In the first study, IOP decreased 55% in the (subtenon) bevacizumab group (from 33.6 ± 4.9 to 15 ± 2 mmHg) and 51% in the control one (from 35 ± 4.0 to 17 ± 3.1 mmHg), while in the second study, IOP decreased 58% in the (intravitreal) bevacizumab group (from an average of 38.4 ± 4.7 to 16 ± 7 mmHg), and 27% in the control group (from 38.5 ± 7.5 to 28 ± 8.4 mmHg). It could be suggested that this result could be explained by some type of methodological difference adopted by these authors. Ranibizumab and conbercept were always intravitreally administered, while bevacizumab’s route of administration varied between studies. The fact that intravitreally administered bevacizumab did not produce significant results in the other (non-statistically significant) studies suggests that this administration method cannot explain the obtained difference, at least not by itself. Moreover, neither the bevacizumab concentrations, nor the surgical technique differed between the five studies. Two main differences arise from these studies. Mahdy et al. (2011) was conducted in individuals with primary congenital glaucoma (PCG). No other included study had a similar population, only two studies with 8 PCG cases in total. As such, no comparisons can be drawn [26, 27]. As for Mahdy et al. (2013), it was conducted in patients with neovascular glaucoma, where anti-VEGF agents are probably the most effective due to the neovascular nature of the disease [28–31]. In fact, this could be perceived as a confounding factor, since anti-VEGF agents may be improving the underlying condition, and only as a consequence, the glaucoma drainage procedure per se. Such hypothesis can be dismantled when future studies find the same statistically effect in other types of glaucoma. Arcieri et al. (2014) was also conducted with NVG patients and using intravitreal bevacizumab, and no significant IOP differences were found. The main difference is that in Mahdy et al. (2013) PRP was given concomitantly with IVB. Another aspect is that Mahdy et al. (2013) provided IVB 2 weeks pre-surgery, while Arcieri et al. did 3 injections, one intra and 2 post-operatively.

We would like to highlight that in Mahdy et al. (2013), PRP has been done in both the bevacizumab and control groups, thus reducing the potential for bias. Nonetheless, this additional intervention could have somehow enhanced bevacizumab’s IOP lowering-effect; however, this theory must be proven, by directly comparing bevacizumab alone with bevacizumab plus PRP.

Finally, neither ranibizumab nor conbercept were found to produce significant differences regarding IOP reduction.

In terms of intra- and postoperative complications, we aggregated the data about the most commonly occurring across all the studies included in this systematic review, with these being choroidal effusion, flat AC, hypotony, and hyphema [32, 33]. The only significant difference between intervention and control groups was found by Zarei et al. (2021), with respect to the higher rate of flat AC in the control group (40% *versus* 10%, p = 0.007). Despite its statistical significance, one´s decision about using (or not) bevacizumab as an adjunctive agent, should not be based solely on this difference, which seems of little clinical relevance and easy resolution.

Our study protocol did not initially address other possible beneficial effects the studied agents could have, apart from the IOP-lowering one. Bevacizumab use led to a significantly lower extension of new AC angle vessels, rubeosis iridis and retinal neovascularization in eyes with NVG. Although these findings have not been consistently associated with a greater IOP reduction after 12-months follow-up, Arcieri et al. (2014) suggested that because of this additional effect, bevacizumab-treated patients tended to need less medication to achieve a good IOP control. Future trials could focus on the potential effect anti-VEGF agents have in the treatment of NVG.

None of the included studies reported significant differences between groups in the reduction of the mean number of glaucoma medications at 12 months. Nonetheless, several studies did not report this parameter.

With respect to mean change in visual acuity, data were too sparse and heterogenous for us to confirm possible clinically meaningful differences between intervention and control groups.

When it comes to the possible limitations of our study, we believe that the following points merit discussion. Despite a comprehensive literature search to evaluate the impact of adjunctive MMC and/or anti-VEGF agents during tube shunt surgery (with 5937 screened references), only 9 RCTs fitted the inclusion criteria. One study in particular raised significant concerns regarding its eligibility, namely Mahdy et. al (2011) which specifically applied to patients younger than 6 years old. The problem would be to introduce a totally different type of population, the pediatric one, in which the intervention, which in this case was bevacizumab, could have a completely different effect from that it could have in an adult population. Other studies in this review included patients with PCG, but of already an adult age. This essentially questions the external validity of these results, that is to say, the extent to which we can generalize their findings, as we cannot be certain if the same significant effect would be found in an adult population.

Regarding the quality of the included evidence, we recognize the wide heterogeneity in patient populations among studies, particularly in terms of age and type of glaucoma. Although this heterogeneity may be relevant, since the results apply to a wide patient population, it may obscure any subgroup effect based on type of glaucoma. Moreover, the overall quality of evidence raised some concerns due to the small sample sizes and the unclear risk of some specific biases. The majority of studies claimed themselves to be “randomized” and “masked”, but then did not explicitly explain the randomization techniques and the procedures they have adopted to mask patients and staff to the allocation group. This raises a question about our ability to detect any incorrectness in the methods and procedures, although we do not believe that this could have had a major effect in the presented results.

Given the shortage of strong and reliable evidence, we searched for other non-RCT scientific papers on this topic. We found one earlier (2004) published review on the effectiveness of mitomycin C in glaucoma drainage devices’ implantation [43], which has come to the conclusion that the benefit of MMC, as well as other antifibrotic agents, is unclear in the context of these types of surgeries. Almost twenty years have passed since this review was published and this subject matter still seems to deserve a more thorough analysis, with more stringent methodologies and reporting all outcomes recommended in the field [44,45].

All studies but one have been conducted with the Ahmed glaucoma valve. Newer devices have meanwhile been introduced which are less bulky and more adapted to the globe’s natural curvature, with more anterior suture fixation points (Ahmed ClearPath) [31] and smaller lumens (PAUL® Glaucoma Implant) [32]. As such, this research topic would perhaps merit a revisit with these newer devices, which may be less prone to fibroblast proliferation [33–35], taking into account the many drawbacks that have been signalled throughout this review.

Another interesting avenue would be to conduct further studies with anti-VEGF agents (with and without concomitant PRP) with NVG patients, given that these were the group of patients where significant benefit seems to exist.

## Conclusion

There is no high-quality evidence to support the use of MMC in tube shunt surgery. As for anti-VEGF agents, specifically bevacizumab, significant benefit seems to exist in neovascular glaucoma patients, especially if combined with PRP. Future studies should be conducted with newer glaucoma devices, which may be less prone to fibrous proliferation.

## Appendix 1

**Search strategies** (last searched on April 27th 2022).

### CENTRAL

#1 (glaucoma drainage implants OR valve OR shunt OR implant OR baerveldt OR ahmed OR paul OR molteno):ti,ab,kw.

#2 (antimetabolites OR mitomycin OR “5 fluorouracil” OR “5 fu”):ti,ab,kw.

#3 (angiogenesis inhibitors OR anti vascular endothelial growth factor OR anti vegf OR aflibercept OR eylea OR bevacizumab OR avastin OR brolucizumab OR beovu OR ranibizumab OR lucentis):ti, ab,kw.

### PubMed

#1 (glaucoma drainage implants OR valve OR shunt OR implant OR baerveldt OR ahmed OR paul OR molteno).

#2 (antimetabolites OR mitomycin OR "5 fluorouracil" OR "5 fu").

#3 (angiogenesis inhibitors OR anti vascular endothelial growth factor OR anti vegf OR aflibercept OR eylea OR bevacizumab OR avastin OR brolucizumab OR beovu OR ranibizumab OR lucentis).

#4 (randomized controlled trial OR controlled clinical trial OR randomized[tiab] randomly[tiab] OR trial[tiab] OR groups[tiab] OR placebo[tiab]).

### Scopus and WoS

#1 ("glaucoma drainage implants" OR implant OR valve OR shunt OR ahmed OR baerveldt OR molteno OR paul).

#2 (antimetabolites OR mitomycin OR "5 fluorouracil" OR "5 fu").

#3 (“angiogenesis inhibitors” OR "anti vascular endothelial growth factor*" OR "anti vegf" OR aflibercept OR eylea OR bevacizumab OR avastin OR brolucizumab OR beovu OR ranibizumab OR lucentis).

#4 (“randomized controlled trial” OR “controlled clinical trial” OR randomized OR randomly OR trial OR groups OR placebo).

### BASEs

(mitomycin “5 fluorouracil” "5 fu" "anti vegf" "anti vascular endothelial growth factor" aflibercept eylea bevacizumab avastin brolucizumab beovu ranibizumab lucentis) glaucoma surgery.
